# The health and economic impact of constructing temporary field hospitals to meet the COVID-19 pandemic surge: Wuhan Leishenshan Hospital in China as a case study

**DOI:** 10.7189/jogh.11.05023

**Published:** 2021-12-04

**Authors:** Yi Cai, Yilin Chen, Linqi Xiao, Sara Khor, Tongzu Liu, Yong Han, Yufeng Yuan, Lin Cai, Guang Zeng, Xinghuan Wang

**Affiliations:** 1 Department of Global Health, Wuhan University School of Public Health, Wuhan, PR China; 2 Department of Urology, Zhongnan Hospital of Wuhan University, Wuhan, Hubei, PR China; 3 Hospital Management Institute, Zhongnan Hospital of Wuhan University, Wuhan, PR China; 4 Department of Hepatobiliary & Pancreatic Surgery, Zhongnan Hospital of Wuhan University, Wuhan, Hubei, PR China; 5 Department of Spine Surgery and Musculoskeletal Tumor, Zhongnan Hospital of Wuhan University, Wuhan, Hubei, PRChina; 6 The Comparative Health Outcomes, Policy, and Economics Institute, School of Pharmacy, University of Washington, Seattle, Washington, USA; 7 Leishenshan Hospital in Wuhan, Wuhan, PR China

## Abstract

**Background:**

In response to the COVID-19 pandemic, two new temporary hospitals were constructed in record time in Wuhan, China, to help combat the fast-spreading virus in February 2020. Using the experience of one of the hospitals as a case study, we discuss the health and economic implications of this response strategy and its potential application in other countries.

**Methods:**

This retrospective observational study analyzed health resource utilization and clinical outcomes data for 2011 inpatients diagnosed with COVID-19 and admitted to Leishenshan Hospital during its 67 days of operation from February 8th to April 14th, 2020. We used a top-down costing approach to estimate the total cost of treating patients at the Leishenshan Hospital, including capital cost for hospital construction, health personnel costs, and direct health care costs. We used a multivariate generalized linear model to examine risk factors associated with in-hospital deaths.

**Results:**

During the 67 days of hospital operation, 19 medical teams comprising of 933 doctors and 2312 nurses were gradually transferred to Leishenshan Hospital from across China. Of the 2011 admissions, 4.5% used intensive care and 2.0% used ventilators. Overall median length of stay was 19 days, and 21 days for patients in the intensive care unit (ICU). The case fatality rate (CFR) was 2.3% overall, 41.8% in the ICU, and 0.4% in general ward (GW). CFRs were 55% and 50% among patients using non-invasive and invasive ventilators, respectively. The mean total cost and direct health care cost were CNY806 997 (US$114 793) and CNY16 087 (US$2288), respectively. Patients admitted to the ICU had much higher direct health care costs, on average, compared to those in the GW (CNY150 415 vs CNY9720, or US$21 396 vs US$1383). The mean direct health care cost per patient with severe or critical diseases was more than five times higher than those with mild or moderate diseases (CNY45 191 vs CNY8838, or US$6428 vs US$1257). Older age, having comorbidities, and critical disease were associated with higher risks of death from COVID-19. Lower health worker to patient ratio (<2.6) was not associated with in-hospital death.

**Conclusion:**

An adequate health workforce were mobilized and deployed to a new temporary hospital. The Leishenshan Hospital increased access to care during the surge in COVID-19 infections, facilitated timely treatment, and transferred COVID-19 patients between GWs and ICUs within the hospital, all of which are potential contributors to lowering the CFR. Patients in the ICU experienced a much higher CFR and a greater burden of health care cost than those in GW. Our results have important implications for other countries interested in constructing temporary emergency hospitals, such as the need for adequate infrastructure capacities and financial support, centralized strategies to mobilize health workforce and to provide respiratory protective devices, and improvement in access to health care.

As of August 4, 2021, almost a year and a half since the lockdown in Wuhan because of the coronavirus disease 2019 (COVID-19) outbreak, there were nearly 200 million confirmed cases of COVID-19 and 4.24 million COVID-19-related deaths across the globe [1]. Many health care systems across the world are overwhelmed with the lack of health care resources to meet the surges of COVID-19 cases, especially intensive care unit (ICU) beds and ventilators during hospitalization [2-9]. More recently, the SARS-CoV-2 delta variant has overtaken the alpha variant in terms of disease transmissibility and hospital risks. Transmissibility increased by 79% since mid-June 2021 and and a higher risk of hospital admission posted greater pressure on hospital beds [10,11]. The number of cases is still increasing exponentially in many countries. The health care systems should be prepared for more variants that may be associated with severe diseases.

Health systems around the world have been faced with the challenge of managing the surge of COVID-19 patients as the waves of infection continue to sweep across the globe. The increased demands for beds and ICUs have been observed in the Lombardy region of Italy, Australia, the United States and Sweden, etc. [5,12-14]. In December 2019, Wuhan, a city in China with a population of 11 million, was the first location in the world facing the COVID-19 outbreak with 50 008 confirmed cases within two months of the first case [15]. The municipal health care system was quickly overwhelmed and immediately reacted by establishing a three-part health care system for early prevention, timely treatment, and quarantine: (1) constructing two new temporary hospitals, Leishenshan and Huoshenshan Hospitals, to meet the surge [16-18]; (2) building Fangcang shelter hospitals for treating mild cases, with lower costs than constructing and running specialty hospitals [19]; and (3) transforming hotels and school dormitories into quarantine sites for isolating suspected cases and monitoring their health status (see Appendix S1 and Figure S2 in the Online Supplementary Document, and **Figure 1**). The case of the Leishenshan Hospital could provide an example to allocate health care resources to meet the need of hospitalization during the pandemic for policy makers.

**Figure 1:**
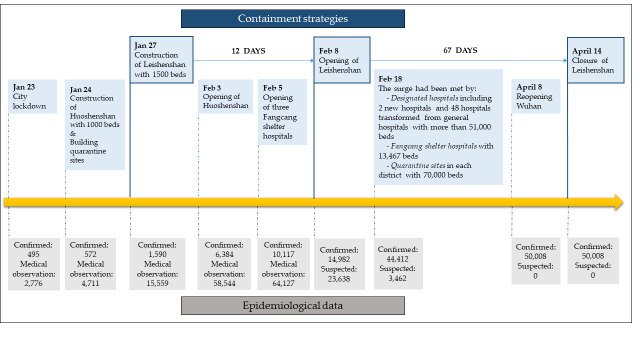
Timeline of the COVID-19 containment in Wuhan.

The strategy of constructing the Leishenshan hospital for treating COVID-19 during the pandemic was built on China’s earlier experience in constructing a new hospital in Xiaotangshan for Severe Acute Respiratory Syndrome (SARS) outbreak in 2003 [19]. This study selected Leishenshan hospital as an example to analyze the health care resource utilization, costs, and clinical outcomes for patients treated at Leishenshan. The study represents a crucial, first-hand experience of the use of newly-constructed temporary hospitals to meet the pandemic surge, which will provide valuable insights for other countries and regions interested in using this strategy to respond to the current and future pandemics.

## METHODS

### Study design and participants

This retrospective observational study included 2011 inpatients discharged from the Leishenshan Hospital (Wuhan, China). Leishenshan Hospital had 1396 hospital beds including 59 ICU beds. It was built in 12 days and was in operation for 67 days from February 8th to April 14, 2020. The last admission was on Mar 19 and the hospital closed after the last patient was discharged on April 14, 2020 [20]. We included all patients who were diagnosed with COVID-19 according to WHO interim guidance, and those who died or were discharged on or before April 14, 2020. Only patients with laboratory-confirmed infections were admitted to the hospital. Patients were discharged once they tested negative for SARS-Cov-2 antigens after two real time reverse transcription polymerase chain reaction tests taken 24 hours apart. Patients who recovered from COVID-19 but had other comorbidities were discharged to general hospitals if their comorbidities needed further treatment. The study was reviewed and approved by the Ethics Commission at Zhongnan Hospital of Wuhan University (2020074).

### Data collection

We extracted de-identified demographic characteristics, clinical outcomes, health care resource use (ie, bed and ventilators), and charge data from the hospital health information system. We collected data on age, sex, insurance type, disease severity according to the national criteria for diagnosis (ie, mild, moderate, severe, and critical cases) [20], comorbidity, admission department (ie, general ward (GW) or ICU), admission date, ventilator use, and the associated treatment outcome, as well as direct health care costs. Insurance type of patients who enrolled in insurance scheme outside Wuhan were classified as other. Comorbidities at the time of admission were classified into five groups according to WHO-China Joint Mission on COVID-19 Report: (1) hypertension: patients with COVID-19 infection concurrent with hypertension but no other comorbidity; (2) diabetes: patients with COVID-19 infection concurrent with diabetes; (3) two comorbidities: patients with COVID-19 infection concurrent with hypertension and diabetes; (4) other: patients diagnosed with COVID-19 infection concurrent with other diseases; and (5) no comorbidity [20]. Treatment outcome was coded as a binary variable (ie, death or discharged alive). The hospital tracked patient expenses—using customary unit costs for hospitals in China—for overnight stays, physical exams, monitoring and laboratory tests (eg, complete blood count, CT, imaging), drug, treatment (eg, cost of ventilator, drug administration, medical procedures), surgery, nursing, medical supplies, and other (ie,, air-conditioning) to obtain cost per patient. We obtained the capital costs for building hospital, supplies and equipment from the government official website and published literature [16,17], and health personnel (including physicians, nurses, therapists, and administration staff) costs from interviews with hospital staff. Transportation costs for transferring medical teams and medical devices to Wuhan were estimated based on the average cost of round-trip to Wuhan by high-speed train. We used patient expenses from the hospital administrative database to estimate the total direct health care costs.

### Statistical analysis

Continuous variables were expressed as means (standard deviation, SD) or medians (interquartile range, IQR) and categorical variables were presented as counts (%). We calculated length of stay (LOS) and case fatality rate (CFR) in overall population and in subpopulations stratified by any ICU use and any ventilator use. We calculated daily health-professional to patient ratios and then computed the median of the daily ratios. To estimate the economic impact of building Leishenshan Hospital in response to this public health emergency, we used a top-down costing approach to calculate the total cost over the entire period for the whole hospital. The total costs included the capital cost for constructing Leishenshan Hospital with 1500 beds, which was estimated at 1.5 times the cost of building Huoshenshan Hospital with 1000 beds at one billion CNY (US$142 million) [17], health personnel costs, transportation costs, and total expenses for treating 2011 patients. Total costs per patient was calculated and all costs were reported in both 2020 CNY and US$ (US$ to CNY: 7.03).

To explore risk factors associated with in-hospital deaths, we used a generalized linear model with the logit link function, accounting for clustering by health professionals. Independent variables were selected based on previous findings and clinical considerations. Recent studies have shown that older age and comorbidities were associated with severe clinical outcomes in adults with COVID-19 [21-23]. We further included sex, insurance type, disease severity, and daily ratio of staff to patient as covariates. All categorical independent variables were coded as dummy variables and the dependent variable was in-hospital death. We set α as 0.05 that was considered statistically significant. We used Stata 13.0 software (StataCorp, College Station, TX, USA) for all statistical analyses.

## RESULTS

### Healthcare resource allocation at Leishenshan

**Figure 2:**
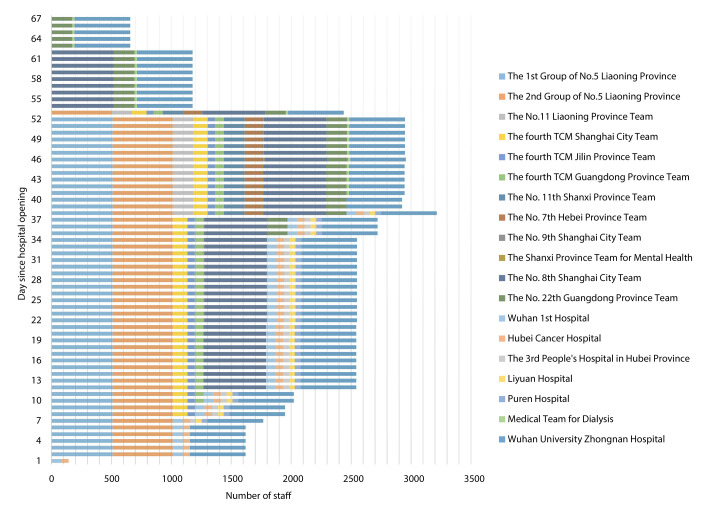
Human resource allocation of 19 medical teams to Leishenshan Hospital.

China’s National Health Commission (NHC) led the allocation of human resources, and the Health Commissions at provincial and municipal levels were responsible for mobilizing and deploying health professionals within their regions to assist Wuhan. A daily medical team deployment system was established to reassemble and send medical teams within 24 hours from provinces across China. A total of 19 voluntary medical teams including 933 doctors and 2312 nurses across China were sent to Leishenshan over the 67-day period (Figure 2). On the first day, two medical teams with 143 health professionals were deployed. The number of health professionals gradually increased and reached the peak at 3214 on day 38. Three medical teams with 657 health professionals remained until the last day of the hospital’s operation. The medical teams composed of health professionals with multi-disciplinary backgrounds, and brought with them PPE and other essential equipment. The number of beds available for use was 1396, including 1337 beds in the GWs and 59 intensive care beds in the ICUs. These beds were gradually put to use as more medical teams joined. The median daily health-professional to patient ratio was 2.6 (IQR: 2.2, 3.5).

### Demographic characteristics of COVID-19 patients

A total of 2011 patients were admitted to Leishenshan Hospital (Table 1). Half of the admitted patients (50.8%) were 60 years old or above, 51.6% were females, and nearly half (42.5%) did not have any comorbidities at baseline. 3.3% and 16.7% of the patients had critical and severe diseases at admission, respectively. The majority of patients (60.2%) were covered by the urban employee health insurance scheme and 14.1% were covered by the resident health insurance scheme. Compared to patients admitted to the GWs (n = 1920), patients admitted to the ICUs (n = 91) were older (mean age: 69.0 vs 57.8 years, SD: 12.1 vs 8.8), and were more often male (64.8% vs 47.6%). More patients in the ICUs had comorbidities at baseline (ICUs vs GW: 95.6% vs 55.7%) and severe disease (severe: 28.6% vs 16.1%; critical: 50.6% vs 1.0%).

**Table 1:** Demographic characteristics of 2011 patients at Leishenshan Hospital*

	Total (N = 2011)	GW (N = 1920)	ICU (N = 91)	Non-invasive (N = 20)	Invasive (N = 26)	ECMO (N = 5)
Age in years, mean (SD)	58.3 (14.7)	57.8 (14.6)	69.0 (12.1)	73.3 (8.8)	69.5 (17.4)	69.8 (12.1)
Age group, years, n (%):						
≤17	7 (0.4%)	7 (0.4%)	-	-	-	-
18-49	521 (25.9%)	517 (26.9%)	4 (4.4%)	-	3 (11.5%)	-
50-59	463 (23.0%)	446 (23.2%)	17 (18.7%)	2 (10.0%)	3 (11.5%)	1 (20.0%)
60-69	577 (28.7%)	551 (28.7%)	25 (27.5%)	6 (30.0%)	5 (19.2%)	2 (40.0%)
70-79	309 (15.4%)	289 (15.1%)	21 (23.1%)	5 (25.0%)	6 (23.1%)	1 (20.0%)
≥80	134 (6.7%)	110 (5.7%)	24 (26.4%)	7 (35.0%)	9 (34.6%)	1 (20.0%)
Sex, n (%):						
Female	1034 (51.6%)	1006 (52.4%)	32 (35.2%)	7 (35.0%)	10 (38.5%)	-
Male	962 (48.4%)	914 (47.6%)	59 (64.8%)	13 (65.0%)	16 (61.5%)	5 (100.0%)
Insurance type, n (%):						
Other	518 (25.8%)	489 (25.5%)	29 (31.9%)	3 (15.0%)	9 (34.6%)	2 (40.0%)
Employee insurance	1210 (60.2%)	1164 (60.6%)	46 (50.6%)	10 (50.0%)	11 (42.3%)	2 (40.0%)
Resident insurance	283 (14.1%)	267 (13.9%)	16 (17.6%)	7 (35.0%)	6 (23.1%)	1 (20.0%)
Comorbidities, n (%):						
None	854 (42.5%)	850 (44.3%)	4 (4.4%)	3 (15.0%)	-	-
Hypertension	447 (22.2%)	420 (21.9%)	27 (29.7%)	4 (20.0%)	6 (23.1%)	2 (40.0%)
Diabetes	108 (5.4%)	98 (5.1%)	10 (11.0%)	1 (5.0%)	1 (3.9%)	-
Hypertension and diabetes	163 (8.1%)	149 (7.8%)	14 (15.4%)	2 (10.0%)	6 (23.1%)	-
Others	439 (21.8%)	403 (21.0%)	36 (39.6%)	10 (50.0%)	13 (50.0%)	3 (60.0%)
Severity, n (%):						
Mild	776 (38.6%)	773 (40.3%)	3 (3.3%)	1 (5.0%)	3 (11.5%)	1 (20.0%)
Moderate	834 (41.5%)	818 (42.6%)	16 (17.6%)	3 (15.0%)	3 (11.5%)	-
Severe	335 (16.7%)	309 (16.1%)	26 (28.6%)	16 (80.0%)	8 (30.8%)	-
Critical	66 (3.3%)	20 (1.0%)	46 (50.6%)	-	12 (46.2%)	4 (80.0%)
Treatment outcomes, n (%):						
Dead	46 (2.3%)	8 (0.4%)	38 (41.8%)	11 (55.0%)	13 (50.0%)	4 (80.0%)
Alive	1965 (97.7%)	1912 (99.6%)	53 (58.2%)	9 (45.0%)	13 (50.0%)	1 (20.0%)

ECMO – extracorporeal membrane oxygenation, ICU – intensive care unit

*Data are mean (SD) or n (%); “-” means no patients in this group.

### Health care utilization and health outcomes

**Figure 3:**
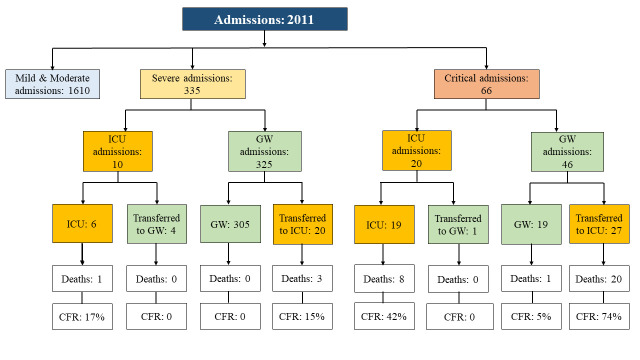
Admission of 2011 patients in general ward (GW) and intensive care unit (ICU).

The number of patients increased from 21 on February 8th to 1240 on March 9th, and then decreased gradually till April 14th. The highest bed occupation rate was 89%. Among the 335 severe patients at admission, 325 (97%) patients were admitted into the GWs, 20 of whom were transferred from the GWs to the ICUs (Figure 3). Among the 66 critical patients at admission, 46 (70%) patients were admitted into the GWs, 27 of whom were eventually transferred to the ICU, and one patient died in the GW due to delayed treatment. This patient had COVID-19 symptoms for 20 days before being admitted to the Leishenshan Hospital (Appendix S2 in the **Online Supplementry Document** shows medical history of the critical patient who died in GW).

Median LOS was 19 days (IQR: 13-25) among 2011 patients (**Table 2**). A total of 20 patients used non-invasive ventilation through a face mask or nasal mask, 26 used invasive ventilation (mechanical ventilation), and five were treated with extracorporeal membrane oxygenation (ECMO). Patients using non-invasive, invasive ventilation, and ECMO had longer LOS on average (median: 42, 27, and 27 days, respectively; IQR: 38-52, 16-43, and 18-38 days) than that of all patients in the ICU. Patients in ICU had a longer LOS than the overall median LOS (median: 21 vs 19 days; IQR: 13-37 vs 13-25 days). Patients with diabetes who used invasive ventilation had the longest LOS of 54 days.

**Table 2:** Length of stay during hospitalization at Leishenshan Hospital*

	Total (N = 2011)	GW (N = 1920)	ICU (N = 91)	Non-invasive (N = 20)	Invasive (N = 26)	ECMO (N = 5)
Length of stay, days	19 (13-25)	18 (13-25)	21 (13-37)	42 (38-52)	27 (16-43)	31 (18-37)
Age group:						
≤17	11 (9-15)	11 (9-15)	0 (0-0)	-	-	-
18-49	15 (10-22)	15 (10-21)	26 (14-35)	-	23 (21-31)	-
50-59	17 (13-23)	17 (13-23)	20 (14-33)	45 (37-52)	18 (16-19)	18 (18-18)
60-69	20 (14-27)	20 (14-27)	21 (8-35)	51 (38-58)	45 (38-50)	32 (3-60)
70-79	22 (15-31)	22 (16-30)	21 (10-37)	50 (43-52)	38 (18-43)	37 (37-37)
≥80	20 (14-31)	20 (14-31)	24 (14-40)	39 (13-40)	18 (13-39)	31 (31-31)
Sex:						
Female	18 (13-25)	18 (13-25)	20 (12-38)	50 (39-52)	30 (16-50)	-
Male	19 (13-26)	19 (13-25)	21 (13-37)	39 (13-52)	27 (17-38)	31 (18-37)
Insurance type:						
Other	18 (12-24)	18 (12-24)	21 (14-32)	50 (52-60)	18 (16-19)	39 (18-60)
Employer insurance	19 (13-26)	19 (13-25)	22 (13-37)	40 (13-58)	37 (18-45)	34 (31-37)
Resident insurance	18 (13-25)	18 (13-24)	36 (9-40)	39 (37-52)	31 (21-43)	3 (3-3)
Comorbidities:						
None	17 (12-23)	17 (12-23)	46 (32-52)	52 (39-52)	-	-
Hypertension	20 (14-26)	20 (14-26)	20 (11-37)	49 (38-59)	28 (12-39)	49 (37-60)
Diabetes	20 (14-26)	19 (14-26)	21 (14-26)	43 (43-43)	54 (54-54)	-
Hypertension and diabetes	20 (14-29)	21 (15-29)	17 (13-21)	26 (13-38)	38 (19-43)	-
Others	19 (13-28)	19 (13-28)	25 (12-36)	45 (13-52)	21 (16-31)	18 (3-31)
Severity:						
Mild	20 (14-27)	20 (14-27)	16 (5-21)	4 (4-4)	18 (16-31)	18 (18-18)
Moderate	15 (11-20)	15 (11-20)	20 (9-26)	-	19 (6-23)	-
Severe	23 (17-33)	24 (17-33)	21 (14-37)	52 (50-52)	44 (20-50)	-
Critical	23 (14-38)	22 (16-40)	27 (13-39)	40 (38-55)	34 (15-39)	34 (17-49)

ECMO – extracorporeal membrane oxygenation, ICU – intensivee care unit

*Data are median (IQR); “-“ means no patients in this group.

**Figure 4:**
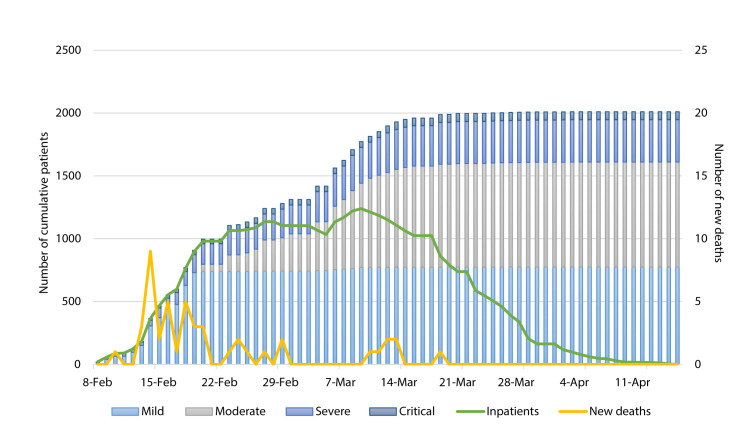
Distribution of admission, inpatient at hospital, and death over time.

The overall CFR was 2.3% among all patients at Leishenshan, while the CFR for patients admitted to ICU (41.8%) was nearly 105 times of that in GW (0.4%) (**Table 1**). Most deaths occurred in the first three weeks between February 8th and March 1st (**Figure 4**). The CFR of patients using non-invasive ventilator (55%) was similar to that of patients using invasive ventilator (50%); the CFR among five patients treated with ECMO was the highest (80%). Among severe and critical patients, we observed the highest CFR among critical patients transferred from the GWs to the ICUs (74%). One critical patient who was admitted to the GW died in three days (**Figure 3**, and Appendix S2 in the **Online Supplementary DocumentI**).

The multivariate generalized linear model showed that older age, having comorbidities, and a diagnosis of critical disease were risk factors of in-hospital death (Table 3). Risk of death was not significantly different among patients with different insurance schemes or with different health professional to patient ratios at admission (**Table 3**).

**Table 3:** Generalized linear model of in-hospital death at Leishenshan Hospital

	Odds ratio	SE	95% CI	*P* value
Age group, years:				
≤17	-	-	-	-
18-49	0.1	0.1	(0-0.5)	0.04
50-59	0.1	0.1	(0-0.4)	0.001
60-69	0.2	0.1	(0.1-0.7)	0.007
70-79	0.4	0.2	(0.1-1.0)	0.05
≥80	Ref	Ref	Ref	Ref
Sex:				
Female	Ref	Ref	Ref	Ref
Male	1.5	0.6	(0.7-3.1)	0.31
Insurance type:				
Other	Ref	Ref	Ref	Ref
Employer insurance	0.4	0.2	(0.2-1.0)	0.06
Resident insurance	0.9	0.5	(0.3-2.5)	0.81
Severity:				
Mild	Ref	Ref	Ref	Ref
Moderate	1.2	0.8	(0.4-4.0)	0.75
Severe	0.8	0.6	(0.2-3.1)	0.80
Critical	44.0	22.0	(16.1-117.1)	0.002
Comorbidities:				
None	Ref	Ref	Ref	Ref
Hypertension	11.3	12.3	(1.4-95.3)	0.025
Diabetes	17.6	21.3	(1.6-189.8)	0.018
Hypertension and diabetes	15.2	17.4	(1.6-143.8)	0.018
Others	27.4	29.1	(3.4-219.2)	0.002
Health-professional to patient ratio	1.3	0.6	(0.6-3.0)	0.53

SE – standard error, CI – confidence interval

### Total cost and cost per patient at Leishenshan Hospital

The number of hospital beds in Leishenshan was 1.5 times of that in Huoshenshan. Based on the expenditures for constructing Huoshenshan hospital (one billion CNY or US$142 million), we estimated the total construction cost of Leishenshan at 1.5 billion CNY (US$213 million). Additionally, each health care professional was paid CNY600 (US$85) per day as a bonus during their shift at Leishenshan, which added up to a total of 87 million CNY (US$12 million) for paying all health personnel. During their services at Leishenshan, health care professionals were paid usual salaries by their affiliated public hospitals, which was not included in the total cost calculation. The total transportation cost for health professionals traveling to Wuhan was estimated to be 3.2 million CNY (US$0.5 million). Finally, including the mean total direct health care costs of 32 million CNY (US$4.6 million), the total estimated cost was 1.62 billion CNY (US$231 million). The mean total cost per patient and the mean total direct health care cost per patient were estimated to be CNY806 997 (US$114 793) and CNY16 087 (US$2288), respectively (**Table 4**).

**Table 4:** Total cost and cost per patient in Leishenshan Hospital

	Total cost		Cost per patient (N = 2011)	
	**CNY**	**US$**	**CNY**	**US$**
Capital cost*	1 500 000 000	213 371 266	745 898	106 102
Personnel cost, bonus†	87 274 200	12 414 538	43 398	6 173
Transportation cost, personnel‡	3 245 000	461 593	1614	230
Direct health care cost	32 350 957	4 601 843	16 087	2 288
Total cost	1 622 870 157	230 849 240	806 997	114 793

*The total capital cost of building two hospitals (Huoshenshan and Leishenshan) was 2.5 billion CNY.

†The bonus for each health care staff was 600 CNY per working day.

‡The average cost of round-trip to Wuhan by high-speed train was 1000 CNY.

**Figure 5:**
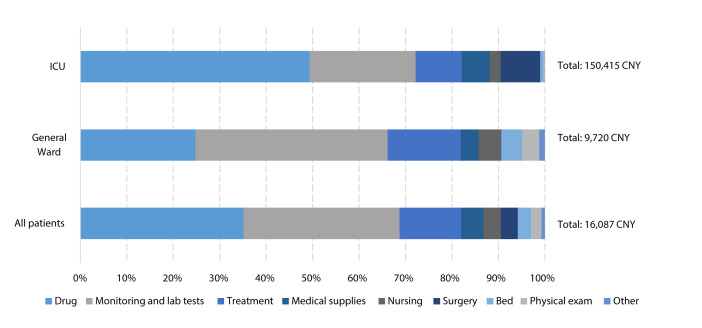
Total mean direct health care costs per person by cost categories.

Patients with various disease severities had different direct health care costs. Patients in the ICUs had direct health care costs 15-fold higher than those in the GWs (mean: CNY150 415 vs CNY9720, or US$21 396 vs US$1383). Comparing to those with mild or moderate diseases, the direct health care cost of patients with severe or critical diseases was more than 5 times higher (mean: CNY45 191 vs CNY8838, or US$6428 vs US$1257). The patient who incurred the highest direct health care cost of over one million CNY (US$142 278) was a patient with critical disease as described in Appendix S2 of the **Online Supplementary Document**. Drug costs and costs associated with monitoring and laboratory tests accounted for most of the total expenses. Specifically, patients in the GWs spent the most on monitoring and laboratory tests, accounting for 41.4% of the total direct health care costs, while patients in the ICUs spent, on average, almost half of the total expenses on drugs (49.4%) (**Figure 5**).

## DISCUSSION

This study provided important empirical evidence of health care resource allocation, as well as the health and economic impacts of building a new temporary specialty field hospital for flattening the pandemic surge in Wuhan. Over the study period, the Leishenshan Hospital was gradually integrated into a three-part health care system with clearer functions. It helped meet the pandemic surge but was very costly. At the Leishenshan Hospital, each patient was being attended by a median of 2.6 health professionals. The overall CFR in Leishenshan over the 67-day period was 2.3% and patients on average spent 19 days in the hospital. An estimated total cost of 1.62 billion CNY (US$231 million) was spent on building and treating patients with COVID-19 in Leishenshan. Our study served as an example of managing the health care needs during a pandemic by constructing a new hospital during the pandemic and provide implications for responding to public health emergencies.

### Implication-1: strengthening infrastructure capacity

Many donor countries are providing infrastructural aid to middle- and low-income countries, such as the economic infrastructure aid by the OECD Development Assistance Committee, South Korea's infrastructure construction projects in ASEAN's developing countries, and Chinese infrastructural aid through the Belt and Road Initiative [24-26]. These countries could also provide aid to construct temporary hospitals in recipient countries with an unmet need of ICU beds during the pandemic.

This paper presented details on how to design a temporary field hospital as well as the allocation of ICU beds (Appendix S1 and Figure S2 in the **Online Supplementary Document**). Through the systemic development of the three-tier medical care system, Leishenshan later functioned as a special hospital for treating patients with critical and severe symptoms. Therefore, more ICU beds should be provided rather than general beds. Consistent with prior studies, our study showed that older people with chronic diseases had higher risk of progressing to severe or critical diseases and a higher CFR than others [21,22,27-29]. More recent evidence showed that vaccination is effective in reducing hospitalization and death [30]. These should be taken into consideration when designing temporary hospitals to meet the surge in future outbreaks.

### Implication-2: mobilization of health workforce

The distribution of health workforce is unequal across the globe. The number of medical doctors per 10 000 population was the highest in Italy (80.13 in 2019), four times of that in China (19.8 in 2017), three times of that in the US (26.04 in 2018), more than 10 time of that in Egypt and South Africa (7.46 and 7.92 in 2019), and even more than 100 times of that many African countries (ie,, 0.77 in Ethiopia in 2018), respectively [31]. In fact, the distribution of health workforce is also unequal at national, provincial, and even county level. In China, for example, high-quality health workforce is centralized in three-tier hospitals located in capital cities [32].

Due to the unequal distribution, it is important to mobilize health workforce to the epicenter. There are two main reasons why China could mobilize health professionals across the country. First, government-established public hospitals form the cornerstone of China’s health care delivery system, where doctors and other health care providers are employees of public hospitals. This setup enabled the rapid health resource mobilization from central and local governments to construct a new hospital to meet the surge. Second, China's central government immediately established the Joint Prevention and Control Mechanism of the State Council to be responsible for coordinating ministries for the health workforce deployment. The experience of Leishenshan provided a few important lessons regarding strategies to mobilize health workforce that may be helpful for policymakers in countries and regions with similar health care systems as China’s.

### Implication-3: adequate financial support while considering efficiency

In the short run, undeniably, it is costly to build a new temporary hospital to be used for 67 days. In our study, we presented a total cost of 1.62 billion CNY spent by 2011 patients (0.8 million CNY per patient). This total consisted of the capital cost (1.5 billion CNY), personnel cost (87 million CNY), transportation cost (3 million CNY), and direct health care cost (32 million CNY). In 2019, the per capita annual income in China excluding tax (ie, “per capita disposable income”) was CNY30 733 (CNY16 021 in rural residents and CNY42 359 NY in urban resident) [33]. The mean total cost was around 50 times and 20 times the annual income of a rural and urban resident, respectively. Although much lower than the mean total cost, the average direct health care cost per case in Leishenshan (CNY16 087) was still high for an individual (ie,, 52% of the average annual income). The average direct health care cost per case in Leishenshan was lower than the estimated cost per case of COVID-19 hospitalization (CNY21 500) in China, according to a recent report by the National Medical Security Bureau [34], but slightly higher than the average annual cost per COVID-19 hospitalization in tier-three public hospitals in China in 2019 (CNY13 664) [35]. In addition, if considering the economic burden on individuals, patients in the ICU could experience a catastrophic expenditure without any financial support. Discharges from ICUs who were severe and critical cases costed CNY150 415 on average. The Chinese government’s decision to use the fiscal budget to cover the out-of-pocket expenditures for all patients protected COVID-19 patients against a medical-related financial crisis and removed financial barriers to care [36]. In the long run, there are a couple of ways to maximize the economic returns after closing the new hospital, such as using it to enhance epidemic preparedness for future outbreaks and for field trainings and drills.

The overall length of stay was long, implying that the efficiency of health resource utilization could be improved. Patients at Leishenshan, especially those in the ICUs, had higher LOS than that in another hospital in Wuhan because of the updated discharge criteria (21 days vs 10 days) [20,37]. The long LOS in the Leishenshan hospital can be explained by the strict criteria of discharging COVID-19 patients. The Trial Version 7 of Diagnosis and Treatment Protocol for COVID-19 required patients in China to meet the following criteria before discharge: (1) body temperature is back to normal for more than three days; (2) obvious improvement in respiratory symptoms; (3) pulmonary imaging shows obvious absorption of inflammation; and (4) nuclei acid test negative twice consecutively on respiratory tract samples such as sputum and nasopharyngeal swabs (sampling interval being at least 24 hours) [20]. If patients meet the first three criteria, they would be discharged to quarantine sites for 14-day observation or general hospitals for treating other conditions. Otherwise, the LOS would be extended until a negative nuclei acid test. A functional designated hospital for COVID-19 patients could be made more efficient in reducing the LOS and relieve the surge in demand for hospital care and ICU care by modifying the discharge criteria and improving referral to shelter hospitals and quarantine sites.

### Implication-4: improving access to health care during the emergency situation

Reducing COVID-19-related mortality and morbidity were the primary goals of constructing the Leishenshan Hospital. The construction of the Leishenshan hospital improved access to inpatient care, which was the key to reducing death risks. First, patients could receive timely treatments when the Leishenshan Hospital opened. We observed that the number of deaths in Leishenshan dramatically decreased when the surge of hospital bed demand had been met since February 20, implying that the death risk decreased when patients could be treated in a timely manner. Only one critical patient died in the GW within three days because of the delay of admitting into the ICU (see Appendix S2 in the **Online Supplementary Document**). Second, the rapid resource mobilization played a key role in ensuring adequate health care resources including health personnel, ventilators, and PPE at the needed sites. This enabled timely essential care, including the use of ventilators to reduce mortality risk when patients had acute respiratory distress syndrome (ARDS). Third, medical teams with expertise in critical medicine were assigned to manage critical patients. There were expert panels providing mobile medical visits to each ICU regularly, and each patient was treated with an individualized diagnosis and treatment plan. High-quality intensive care may have increased their survival chance.

There are some limitations in our study. First, this study described the experience of one hospital in Wuhan and the empirical findings may not be generalizable to other hospitals or regions in China. Second, since a few patients were transferred from other hospitals or were discharged to other hospitals for treating comorbidities, the mean LOS and the direct health care cost estimates for COVID-19 patients were likely conservative. Third, some costs were not included in the total cost, such as donations of some medical equipment (ie,, ventilators, negative pressure ambulance, and personal protective equipment), and regular salaries of health professionals paid by their employers. Thus, in this study, the total cost was likely under-estimated. Last, the health care systems are different across countries and regions. The Leishenshan hospital was operated in a three-tier health care system, which included the Fangcang shelter hospitals for treating milder cases and quarantine sites. Other countries and regions will need to adapt their pandemic response strategies to their own health care systems.

## CONCLUSION

This is the first empirical study to describe the health care resource allocation strategies, health outcomes, and costs related to building a new temporary hospital in China to respond to the COVID-19 pandemic. China's experience with the construction of the Leishenshan Hospital demonstrated the benefit of building temporary hospitals during the early phase of an outbreak. The Leishenshan Hospital increased access to care during the surge in COVID-19 infections, facilitated timely treatment, and transferred COVID-19 patients between GWs and ICUs within the hospital, all of which are potential contributors to lowering the CFR. In addition, it freed up resources in other hospitals to care for patients with other conditions and needs, such as acute care or surgeries, potentially reducing overall comorbidity and death. As a preparedness strategy for preventing substantial loss of lives, countries and regions with similar health care system and the capacity to build temporary hospitals should adapt this strategy to prevent overwhelming the existing health systems and exhausting resources needed for other patients. For example, countries with decentralized health systems can establish public health emergency teams at the national or federal level for rapid pandemic response. Several aspects of the construction design and the efficiency of Leishenshan could also be improved in the future through several actions: (a) allocating a higher proportion of ICU beds for treating severe and critical patients, (b) developing specific discharge criteria to reduce the LOS in temporary specialty hospitals, and (c) empowering multiple types of medical staff to both treat patients during the outbreak and provide training and drills to prepare for new outbreaks.

## Additional material


Online Supplementary Document
