# Supplementary material for: The health and economic impact of constructing temporary field hospitals to meet the COVID-19 pandemic surge: Wuhan Leishenshan Hospital in China as a case study
Source: J Glob Health. 2021 Dec 4;11:05023. doi: 10.7189/jogh.11.05023 (PMC8645218; doi:10.7189/jogh.11.05023)
Supplement: Online Supplementary Document [file jogh-11-05023-s001.pdf]

Appendix SI. Strategies of establishing a three-part healthcare system for containing COVID-19 in Wuhan

Part -1: Building designated hospitals

During the early outbreak of COVID-19, the number of confirmed cases increased exponentially and quickly overwhelmed the healthcare system in Wuhan. In order to relieve the pressure on hospital care, the Chinese government built two new designated hospitals. The first new designated was a 1000-bed hospital in Wuhan, the Huoshenshan Hospital, for treating COVID-19 patients which was built on January 24. However, the number of confirmed cases tripled from 495 to 1,590 between January 23 to 27. The government immediately started to build another new hospital, the Leishenshan Hospital with 1,500 beds, in Wuhan city. The construction design of Leishenshan was improved based on Huoshenshan's design. Huoshenshan and Leishenshan Hospitals started receiving patients on February 3 and February 8, respectively. Meanwhile, 48 of these hospitals completed their reconstruction and were reassigned as designated hospitals for treating COVID-19 as of February 18, 2020.

Part - 2 & 3: Building Fangcang shelter hospitals and quarantine sites

Between January 27 and February 5, the number of confirmed cases increased by six times, and doubled within five days. Since most of the confirmed patients were mild and moderate, Wuhan started to build Fangcang shelter hospitals for isolating and treating patients, which had lower investments and direct healthcare costs than building designated hospitals, and could be built in 24 hours for each. On February 5, three Fangcang shelter hospitals had been opened for accepting mild and moderate patients, and designated hospitals only accepted severe and critical patients in order to save more beds. Additionally, Wuhan had transformed hotels and school dormitories into quarantine sites, providing approximately 70,000 beds for isolating suspected cases and monitoring their health status regularly by community health professionals.

By February 18, a three-part healthcare system for isolating and treating COVID-19, including designated hospitals for severe and critical patients, Fangcang shelter hospitals for mild and moderate patients, and quarantine sites for suspected cases, was established in Wuhan. The number of hospital beds in designated hospitals exceeds the number of confirmed cases by 51,000 beds at the epidemic peak in Wuhan. On February 20, Wuhan officials announced that the surge in demand for hospital beds was met.

Figure S1. Layout of Leishenshan Hospital

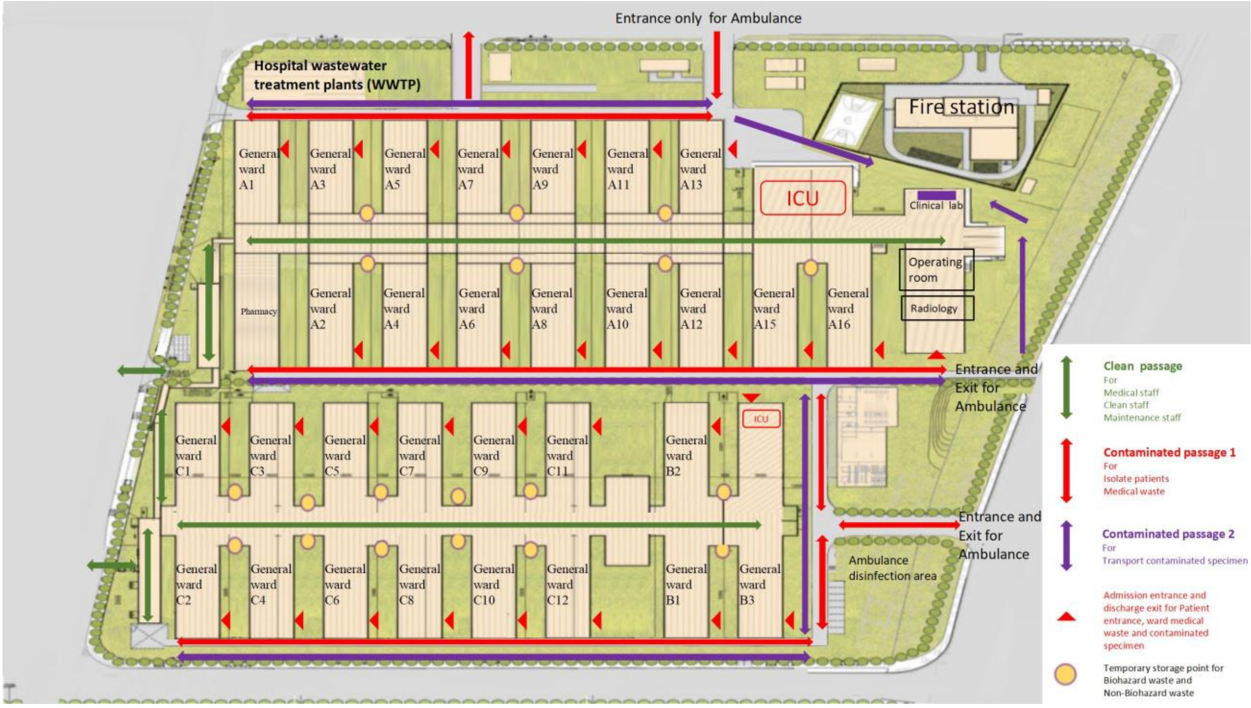

Appendix S2.. Case reports of critical patients in Leishenshan Hospital

Critical patient: Female, 84 years old, GW, no comorbidity

This patient, admitted on February 19th, was the only critical patient who was not admitted to the ICU and died within three days at the hospital. She had a fever and cough for 20 days before she was admitted to the Leishenshan hospital. She was diagnosed with respiratory failure during admission. She received medications, nutritional support, and non-invasive ventilation treatment. During the hospitalization, she was rescued twice and died during the second time. After the patient died, an expert panel including eight physicians who directly took care of her reviewed the process of her hospitalization during a death case discussion and documented their summary in the death case discussion sheet.

Critical patient: Male, 68 years old, ICU, with hypertension, hyperlipidemia, pulmonary embolism, and cardiac pacemaker

On February 10th, the patient was admitted to the hospital after testing positive for COVID-19. He received a CT scan and was admitted to the ICU. During his hospitalization, the patient received multiple procedures, such as stent placement, hemodialysis, bronchoalveolar lavage, bone grafting, mechanical ventilation, and extracorporeal membrane oxygenation (ECMO). He was given different types of antibiotics, antiviral medicines, Chinese medicines, and other drugs for treating various complications. On April 6th, the patient died after being hospitalized for 56 days. His total healthcare cost was 1,639,778 CNY (233,254.3 USD).
